# Proteomic Profiling of Acute Promyelocytic Leukemia Identifies Two Protein Signatures Associated with Relapse

**DOI:** 10.1002/prca.201800133

**Published:** 2019-02-04

**Authors:** Fieke W. Hoff, Chenyue W. Hu, Amina A. Qutub, Yihua Qiu, Marisa J. Hornbaker, Carlos Bueso‐Ramos, Hussein A. Abbas, Sean M. Post, Eveline S. J. M. de Bont, Steven M. Kornblau

**Affiliations:** ^1^ Department of Pediatric Oncology/Hematology Beatrix Children's Hospital University Medical Center Groningen University of Groningen Groningen 9713 The Netherlands; ^2^ Department of Bioengineering Rice University Houston TX 77030 USA; ^3^ Department of Leukemia The University of Texas MD Anderson Cancer Center Houston TX 77030‐4009 USA; ^4^ The University of Texas Graduate School of Biomedical Sciences at Houston Houston TX 77030 USA; ^5^ Department of Hematopathology The University of Texas MD Anderson Cancer Center Houston TX 77030 USA; ^6^ Hematology and Oncology Fellowship Program Cancer Medicine Division The University of Texas MD Anderson Cancer Center Houston TX 77030 USA; ^7^ Department of Biomedical Engineering University of Texas San Antonio San Antonio TX 78429 USA

**Keywords:** acute myeloid leukemia, acute promyelocytic leukemia, leukemia, proteomics reverse phase protein array

## Abstract

**Purpose:**

Acute promyelocytic leukemia (APL) is the most prognostically favorable subtype of Acute myeloid leukemia (AML). Defining the features that allow identification of APL patients likely to relapse after therapy remains challenging.

**Experimental Design:**

Proteomic profiling is performed on 20 newly diagnosed APL, 205 non‐APL AML, and 10 normal CD34+ samples using Reverse Phase Protein Arrays probed with 230 antibodies.

**Results:**

Comparison between APL and non‐APL AML samples identifies 8.3% of the proteins to be differentially expressed. Proteins higher expressed in APL are involved in the pro‐apoptotic pathways or are linked to higher proliferation. The “MetaGalaxy” approach that considers proteins in relation to other assayed proteins stratifies the APL patients into two protein signatures. All of the relapse patients (*n* = 4/4) are in protein signature 2 (S2). Comparison of proteins between the signatures shows significant differences in relative expression for 38 proteins. Protein expression summary plots suggest less translational activity in combination with a less proliferative character for S2 compared to signature 1.

**Conclusions and Clinical Relevance:**

This study provides a potential proteomic‐based classification of APL patients that may be useful for risk stratification and therapeutic guidance. Validation in a larger independent cohort is required.

## 1 Introduction

Acute promyelocytic leukemia (APL) accounts for 10–12%^1^ of all acute myeloid leukemia (AML) cases and is the most prognostically favorable subtype of AML. Epidemiologically, it is more common in younger patients and those with a Hispanic background.^2^ APL, historically classified under the French–American–British classification system as M3, is cytogenetically defined by the reciprocal translocation t(15;17)(q24;q21) between the promyelocytic leukemia (PML) gene on chromosome 15 and the transcription factor retinoic acid receptor α (RARα) on chromosome 17, which is present in more than 95% of APL patients.^3^, ^4^, ^5^, ^6^ This genetic cancer driver results in the PML‐RARα fusion oncogene and chimeric protein, which interferes with the RARα signaling, thus blocking cell differentiation of the myeloid progenitor and stimulating aberrant self‐renewal.^3^, ^7^ In 5% of the patients, other chromosomal rearrangements such as complex translocations or insertions drive the APL, including ZBTB16–RARA, NUMA1‐RARA, and NPM1–RARA. Current synergistic treatment with the all‐trans‐retinoic acid (ATRA) vitamin A derivative, which induces terminal differentiation of promyelocytes, in combination with arsenic trioxide (ATO), which binds to PML and accelerates degradation of the PML‐RARα fusion protein resulting in partial differentiation and induction of apoptosis,^4^, ^6^, ^8^ produces remission in over 90% of cases. However, 5–20% of the patients will relapse and not all can be salvaged by additional therapy or stem cell transplantation. Therefore, a clinical need exists to identify those who are likely to relapse and to find novel therapeutics to add into the therapeutic regimen.^4^

Previously, we demonstrated that AML could be classified by recurrent patterns of protein expression using Reverse Phase Protein Arrays (RPPA),^9^, ^10^, ^11^ a highly sensitive and reproducible, high‐throughput technique that provided prognostic information and suggested targets for drug development.^9^, ^10^, ^11^, ^12^, ^13^ Gene expression profiling (GEP) has revealed recurrent patterns of gene expression,^14^ but has the limitation that messenger RNA transcript expression correlates with protein abundance for less than 50% of genes and does not reflect post‐translational modifications.^15^, ^16^, ^17^ In this study, we aimed to define the protein expression patterns of APL with the additional goals of determining how they compare to those of AML and to see if certain patterns would be prognostic for outcome, and thereby suggest novel therapy targets. Herein, we demonstrate that even though APL and AML share significant similarities in the expression levels of many proteins, there are distinct protein expression patterns that may explain differences in response to therapy. In addition, we found that protein expression patterns distinguished a subset of APL patients as high‐risk for relapse.

## 2 Experimental Section

### 2.1 Patient Population

Peripheral blood and bone marrow samples were collected from 20 newly diagnosed APL patients and 511 newly diagnosed AML patients that were evaluated at The University of Texas MD Anderson Cancer Center between September 1999 and March 2007. Samples were collected prior to induction therapy in accordance with institutional IRB policies. Informed consent was obtained in accordance with the *Declaration of Helsinki* and applicable local and state laws. Because it was observed that some protein expression patterns were exclusively present in cryopreserved cells,^9^ the analysis was restricted to the 205 non‐APL AML fresh samples to work with more native patterns. For the APL cases, a mixture of cryopreserved (*n* = 9) and fresh samples (*n* = 11) was used due to the sample size. The APL patient demographics are described in Table 1 and those of the AML cases in Table S1, Supporting Information. APL patients had a median age of 42.5 years, which is representative for APL. Seventeen patients had the t(15;17) translocation, while the other three were confirmed to be APL by the PML oncogenic domains (POD) test or by PCR. All but one of the patients were treated with ATRA, including 14 in combination with ATO alone (*n* = 8) or with gemtuzumab (*n* = 5) or idarubicin (*n* = 1) if high risk features were present, another five received ATRA with gemtuzumab (*n* = 1) or idarubicin (*n* = 2) or both (*n* = 1), and one received only liposomal ATRA. One patient was treated only with idarubicin and cytosine arabinoside. All but one (95.0%) achieved complete remission (CR), with one early death due to hemorrhage. Four patients relapsed (two received ATRA plus ATO, one ATRA plus gemtuzumab, and one ATRA plus idarubicin) and one patient died of concurrent metastatic breast cancer with bone marrow infiltration and was therefore excluded from the outcome analysis. Eighty‐five percent (*n* = 17) were still alive at the end of follow‐up (range 83–437 weeks).

**Clinical Relevance**

APL is a very different form of AML, mostly driven by the reciprocal translocation t(15;17)(q24;q21), and in contrast to other subtypes of AML has a relatively favorable prognosis. This suggests a much greater homogeneity of leukemogenesis in APL than in other forms of AML. Although, the cure rate of APL is high with a complete remission rate of more than 90%, 5–20% of the patients will relapse and not all can be salvaged. A clinical need exists to identify those who are likely to relapse and to find novel therapeutics to add into the therapeutic regimen. Since the combined consequences of genetic and epigenetic events culminate in a net effect manifested at the protein level, we applied the RPPA methodology to determine protein expression levels in 20 APL patients. We identified the existence of two protein signatures based on recurrences in protein expression patterns. Protein signatures were associated with relapse, suggesting that high‐risk APL patients could potentially be identified based on their proteomic profiles. Identification of aberrantly expressed proteins in those patients could then be used in the process of risk‐stratification and to select drugs that target those proteins in combination with standard therapy in high‐risk patients.

**Table 1:** Demographics and clinical characteristics of 20 newly diagnosed APL patients

**Variable category**	**All**	**Signature 1**	**Signature 2**	***p***
**Number of cases (*n*)**	20	7	13	
**Male**	55.0%	57.1%	53.8%	**1.000**
**Age, y**				
Mean	42.0	44.1	41.0	**0.552**
Median	42.5	48.3	39.2	
Maximum	71.0	71.0	67.7	
Minimum	14.8	14.8	18.0	
**Cytogenetics**				
t(15;17)	85.0%	75.7%	84.6%	**0.523**
Diploid^a)^	10.0%	14.3%	7.7%	
Unknown^a^	5.0%	0.0%	7.7%	
**FLT3**				
ITD	30.0%	28.6%	30.8%	**1.000**
D835	20.0%	28.6%	15.4%	
Unknown	10.0%	0.0%	28.6%	
**NPM1**				
Mutant	15.0%	0.0%	23.1%	**1.000**
Wildtype	50.0%	28.6%	61.5%	
Unknown	35.0%	71.4%	15.4%	
**Response**				
CR	95.0%	100.0%	92.3%	**1.000**
Early death	5.0%	0.0%	7.7%	
**Event**				
Yes	25.0%	0.0%	38.5%	**0.106**
**Alive**				
Yes	85.0%	100.0%	76.9%	**0.521**

a Those patients were confirmed to be APL by the POD test or PCR.

### 2.2 RPPA Methodology

RPPA were performed on samples from patients with APL and AML. The methodology and validation of this technique are fully described in previous publications.^13^, ^18^, ^19^ Briefly, samples were enriched for leukemic cells by performing Ficoll separation to yield a mononuclear fraction followed by CD3/CD19 depletion to remove contaminating T and B cells, if they were calculated to be >5% based on the post‐Ficoll differential. All samples were normalized to a concentration of 1 × 10^4^ cells mL^−1^ and printed in five (1:2) serial dilutions onto slides along with normalization and expression controls. Slides were probed with 230 strictly validated primary antibodies and a secondary antibody to amplify the signal, and finally a stable dye was precipitated. This included antibodies targeting 169 different proteins, together with 52 antibodies against phosphorylation sites, 6 targeting Caspase and Parp1 cleavage forms and 3 targeting histone methylation sites. Antibodies were selected based on interest or suspected interest based on their reported function, or based on prior reports suggesting a relationship to leukemogenesis. A table of the manufacturer, antibody name, and primary and secondary antibody dilution can be found in Table S2, Supporting Information. The stained slides were analyzed using MicroVigene software (Version 3.4, Vigene Tech, Carlisle, MA) to produce quantified data.

### 2.3 Data Normalization and Processing

*SuperCurve* algorithms^20^ were used to generate a single value from the five serial dilutions. Loading controls^21^ and topographical normalization procedures^22^ were performed to account for protein concentration and background staining variations. All samples were printed in replicate, and the average expression level of each replicate was used as a single expression level. Protein expression levels were shifted relative to the median of the normal CD34+ bone marrow samples.

### 2.4 Computational Analysis

The computational analysis schema was done using the “MetaGalaxy” analysis as previously fully described by the group.^10^, ^11^, ^12^ Briefly, the 230 proteins were first divided into 31 “protein functional groups” (PFGs) based on their known functions or pathway membership from the existing literature or based on strong associations within the dataset. The allocation of antibodies into their PFG is listed in Table S2, Supporting Information. Various “protein clusters” that expressed similar correlated protein expression patterns were identified within each PFG for the AML patients.^11^ To identify whether each new APL case belonged to one of the AML‐defined protein clusters, or to a novel protein cluster, linear discriminant analysis^23^ was performed. Next, the 205 AML patients were clustered based on a compilation of their protein cluster membership. This identified 11 “protein constellations”: strong recurrent correlations between protein clusters. A group of patients with similar patterns of protein constellations were defined and 13 “protein expression signatures” identified. To determine if protein expression patterns in APL were similar to, or distinct from, those of AML, Random Forest^24^ decision tree was applied to predict constellation membership of the newly formed APL protein clusters and signature membership for the 20 APL cases. Correlations between signatures and clinical features were assessed using the Fisher's exact test for categorical variables and the Kruskal–Wallis test for continuous variables. Survival curves were generated using the Kaplan–Meier method. Individual proteins were compared between the APL and AML samples and between the APL signatures using the Student's *t*‐test with Bonferroni correction (*p* < 0.05). All the statistical tests and plots were generated in R (Version 0.99.484 – 2009–2015 RStudio, Inc.).

### 2.5 Pathway Analysis

Differentially expressed proteins between AML and APL were analyzed for pathway enrichment by utilizing BP, KEGG, and Reactome compendiums, using over‐representation analysis with a hypergeometric distribution; significance was considered for *p* < 0.05. STRING software (String 10.5; available from http://string-db.org) was used to determine protein associations between the differentially expressed proteins between the signatures.

### 2.6 Immunohistochemistry

Formalin‐fixed paraffin‐embedded bone marrow biopsies corresponding to samples that were analyzed on the RPPA were deparaffinized in xylene and rehydrated in an alcohol gradient. Antigen retrieval was performed using citric acid buffer 10 mm sodium citrate, 0.05% Tween 20 (pH 6.0) in a steam chamber for 45 min. Slides were incubated in a 3% hydrogen peroxide/methanol solution to deactivate endogenous peroxidase and then incubated with a primary antibody against HNRNPK (Abcam, ab18195, primary dilution 1:3000) at 4 °C overnight in a humidity chamber. Biotinylated anti‐mouse secondary antibody was added at room temperature for 30 min, and antibody–protein interactions were visualized with the Vectastain Elite ABC and DAB peroxidase substrate kits. Counterstains were performed with nuclear fast red.

## 3 Results

### 3.1 Leukemic Cells of APL and Non‐APL AML Patients Express Distinct Protein Expression Levels

Expression levels were measured in our cohort of AML and APL patients relative to normal CD34+ bone marrow samples and compared. The significance of differences was assessed using the Student's *t*‐test with Bonferroni adjusted *p*‐value (0.05/230 = 0.000217). This resulted in 19 (8.3%) proteins (Table 2) that were differentially expressed between AML and APL. To investigate which pathways were enriched among the 19 proteins that were differentially expressed between APL and AML, pathway enrichment analysis was performed. We identified that pathways involved in apoptosis and cell development were the most strongly enriched. The seven proteins that contributed to those pathways included ASNS, BCL2, BCL2L1, CDKN2A, DIABLO, YWHAZ, and ZNF346, which were all elevated in APL compared to AML, except for ZNF346 which was higher in AML. Corresponding enriched pathways are summarized in Table S3, Supporting Information.

**Table 2:** Nineteen differentially expressed proteins (log 2 scale) between the APL and non‐APL AML patient samples. Median expression levels are relative to the healthy CD34+ cells. *p*‐Values are Bonferroni adjusted (alpha < 0.05). Proteins are listed alphabetically

	**Higher APL**				**Higher AML**		
**Protein**	**Median AML**	**Median APL**	***p***	**Protein**	**Median AML**	**Median APL**	***p***
ASNS	−0.210	0.395	0.032	ATG7	0.406	−0.011	0.001
BCL2	−0.489	0.814	0.000	EIF4EBP1.pS65	0.590	−0.019	0.001
BCL2L1	−0.065	0.391	0.005	GSKA_B	0.153	−0.242	0.003
CDKN2A	−0.215	0.642	0.000	GSKA_B.pS21_9	0.021	−0.324	0.001
DIABLO	0.019	0.642	0.040	INPPL1	0.395	0.116	0.012
IGFBP2	−0.654	0.756	0.000	KDR	0.013	−0.181	0.033
PIK3CA	−0.024	0.346	0.003	PTPN11	0.317	0.125	0.016
RPS6.pS240_244	0.019	1.105	0.002	RPS6KB1.pT389	1.522	−0.160	0.000
YAP1.p	−0.449	0.062	0.000	ZNF346	0.075	−0.025	0.010
YWHAZ	−3.067	−0.005	0.000				

**Figure 1:**
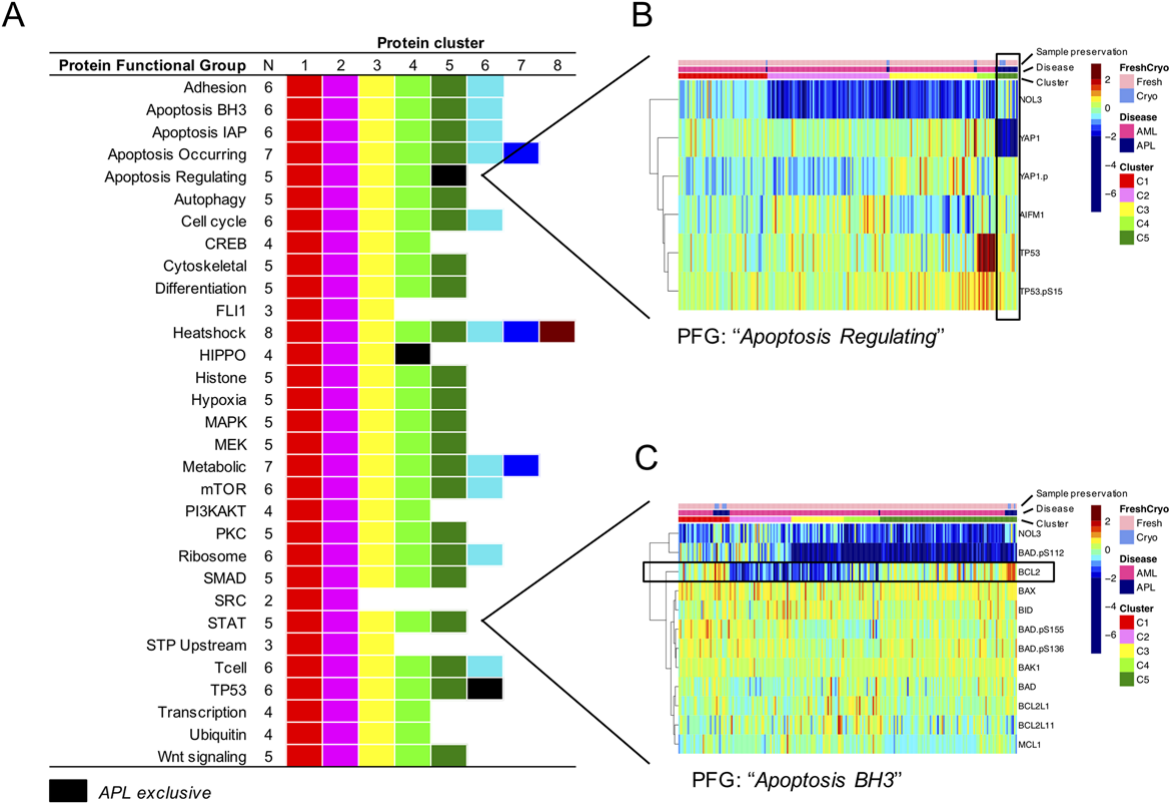
A) The optimal number of protein clusters for each protein functional group. The annotation bar on top of the heatmaps (“Disease”) shows non‐APL AML patients in pink and APL patients in blue. B) Protein cluster C5 (dark green) of the PFG “Apoptosis Regulating” was exclusive to APL patients and is outlined by the black box. C) For the PFG “Apoptosis BH3,” APL cases were intermixed with the non‐APL AML cases in protein cluster C1 and C5.

Next, protein antibodies were divided into 31 PFGs based on their known pathway membership and functionality from the literature to analyze proteins in the context of other functionally related proteins. As previously published,^9^, ^10^, ^11^, ^12^ the Progeny Clustering algorithm^25^ was applied to each group of proteins and this identified that within each PFG, subgroups of AML patients could be recognized that expressed similar (correlated) patterns of protein expression of key protein members of that group. These correlated patterns were defined as a protein cluster. In this study, we integrated our cohort of 20 APL patients into the 154 existing AML protein clusters and identified that for almost all PFGs, the APL patients fell into one of the clusters already identified as occurring in AML. Only for the PFGs “Apoptosis regulating”, “HIPPO,” and “TP53” did we observe that some APL cases had expression patterns that were distinct from the AML cases. These three clusters were primarily formed by altered expression of YAP1 (relatively low in APL) and 14‐3‐3 protein zeta (YWHAZ) (higher in APL compared to AML), and these are both members of the HIPPO and TP53 PFG, again highlighting differences between APL and AML in apoptosis‐regulating pathways. Although, protein clusters in the “Apoptosis BH3” PFG were not exclusively formed by APL (Figure 1C), the APL patients were all clustered in protein cluster 1 and 5, two clusters that showed higher BCL2 compared to the healthy CD34+ samples, whereas protein clusters 2, 3, and 4 had all lower BCL2 compared to normal. Figure 1 provides an overview of the previously established AML clusters in addition to the newly defined APL‐exclusive PFG clusters (Figure 1A, black fill indicates APL‐exclusive clusters). It also provides an example of where the APL cases form an exclusively new PFG cluster (Figure 1B) and an example of where the APL cases are intermixed with the AML cases (Figure 1C).

### 3.2 Global Recurrences in Protein Expression Could Not Identify APL from AML

**Figure 2:**
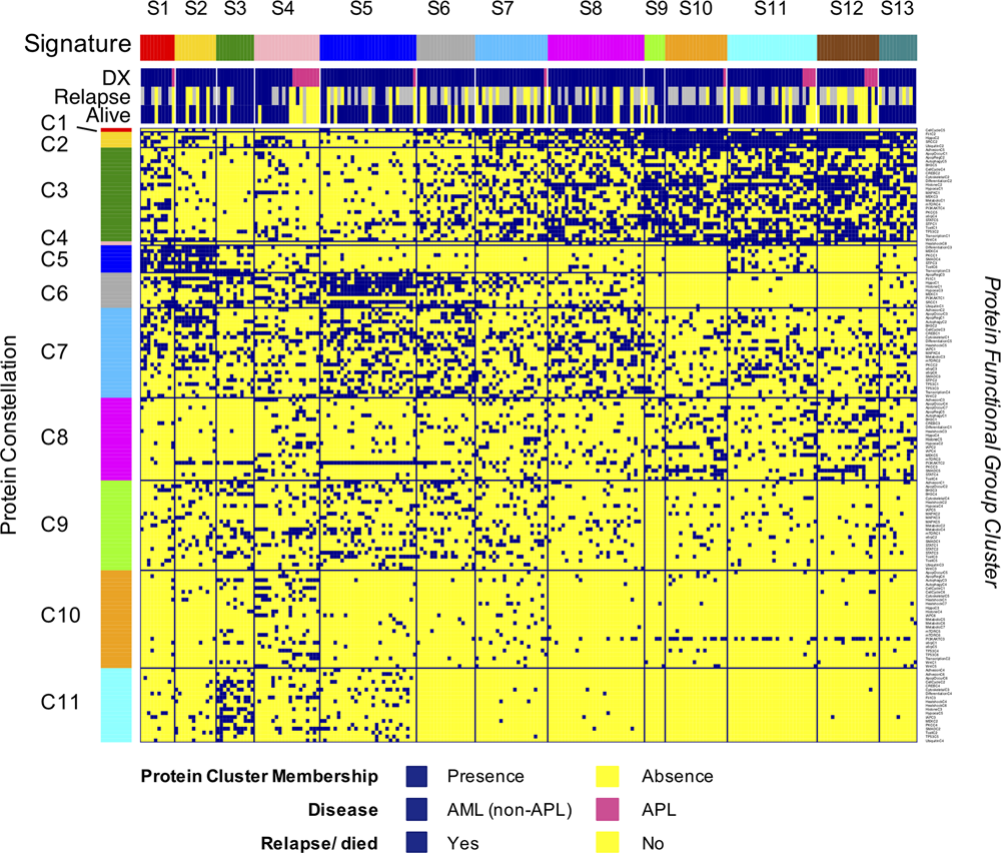
Binary block clustering of the protein clusters identified 13 protein constellations (horizontally) that formed 12 protein‐expression signatures (vertically). A vertical line represents one individual patient. A row represents one protein cluster. Annotations are included at the top (“DX”) and show whether a patient was diagnosed with APL (pink) or another classification of AML (dark blue).

We next looked to see if recurrent protein patterns also occurred in APL and if those were similar to, or distinct from, those seen in AML. Previously, we combined the AML patients and protein clusters into one binary matrix by coding each patient as 1 or 0 for their protein cluster membership which we called the “MetaGalaxy”: 1 if a member, 0 if not a member. Block clustering identified highly correlated protein clusters and recognized an optimal number of 13 protein constellations. These are protein clusters from different PFGs that tightly co‐cluster with each other. Subsequently, patients that expressed similar patterns of protein constellations could be characterized by 13 protein expression signatures. Here, we applied Random Forest decision trees to predict constellation membership for the three new protein clusters (*Apoptosis Regulating C5, HIPPO C4, TP53 C6*) and to predict protein signature membership for the 20 APL patients. Figure 2 shows the extended version of “MetaGalaxy,” showing AML patient in dark blue and APL patients in pink (annotation bar “DX”). Within the 13 protein signatures, we observed seven signatures that included some APL patients, with several APL patients found in signatures 4, 11, and 12, and individual cases appearing in signatures 1, 5, 7, and 10. This suggests that in contrast to the differences that exist in the individual pathways, the global protein expression patterns of APL patients are comparable to those of non‐APL AML cases.

### 3.3 Co‐Clustering Revealed APL Protein Expression Signatures Correlated With Outcome

**Figure 3:**
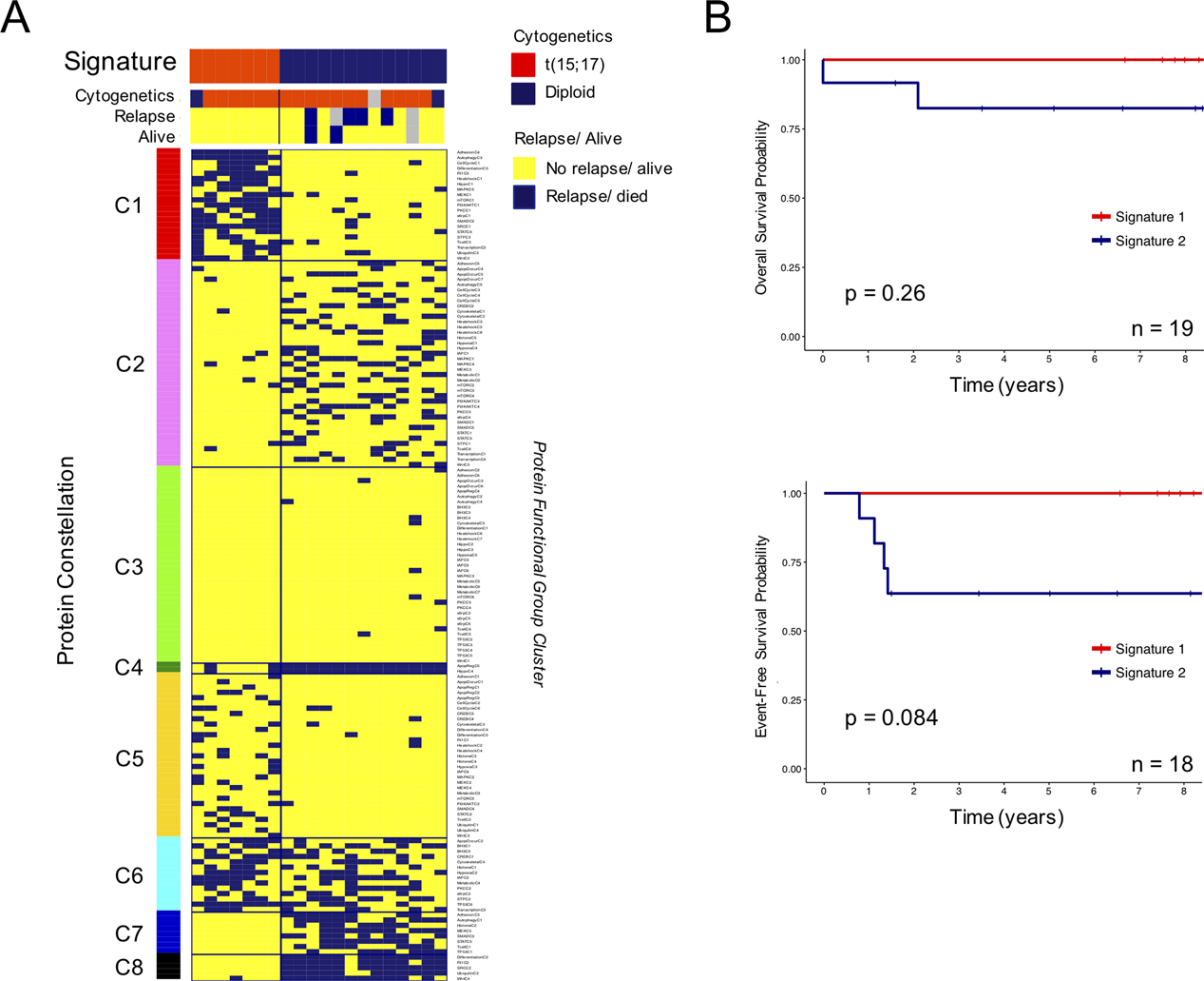
A) Co‐clustering of protein clusters from all protein functional groups for our cohort of APL patients. Columns indicate individual patients and rows represent the protein clusters. B) Overall survival and complete remission duration are shown stratified by signature. Colors indicate signature membership as annotated on the “MetaGalaxy”(“Signature”).

When we considered our APL cohort separately, we were able to identify two distinct protein expression signatures (S1 and S2) using the “MetaGalaxy” approach that yielded prognostic information (Figure 3). Again, protein signatures were defined as a group of patients that expressed similar patterns of protein constellations. The robustness of the signatures was confirmed by the traditional unsupervised hierarchical clustering approach and by consensus clustering; each method identified two clusters formed by the same clusters of patients (Figure S1, Supporting Information). S1 showed strong associations with constellation 1 and 6, whereas S2 included patients that expressed patterns seen in constellation 4, 6, 7, and 8. Survival analysis showed that all of the patients that relapsed were in S2, which suggests a favorable prognosis for patients that express protein patterns associated with S1. However, due to the high overall survival (OS) of 89.5% (*n* = 17/19), the low relapse rate (*n* = 4/18, 22.2%), and the small number of patients in our cohort, OS (*p* = 0.258) and CR duration (*p* = 0.084) were not statistically significantly different between the two signatures. Other variables that were associated with protein signatures were the number of promyelocytes in the bone marrow (low in S1, median 47% vs 71% overall; *p* = 0.070) and fibrinogen levels (higher in S1, median 235 mg dL^−1^ vs 159 mg dL^−1^; *p* = 0.052). Although we had small numbers, all NPM1 mutated patients (*n* = 3/3) were within S2. No association with the FLT3‐ITD mutation status was observed. Cryopreservation did not seem to affect the protein expression patterns, and there was no signature dominated by cryopreserved samples.

### 3.4 APL Patient Signatures Express Dissimilar Protein Patterns

**Figure 4:**
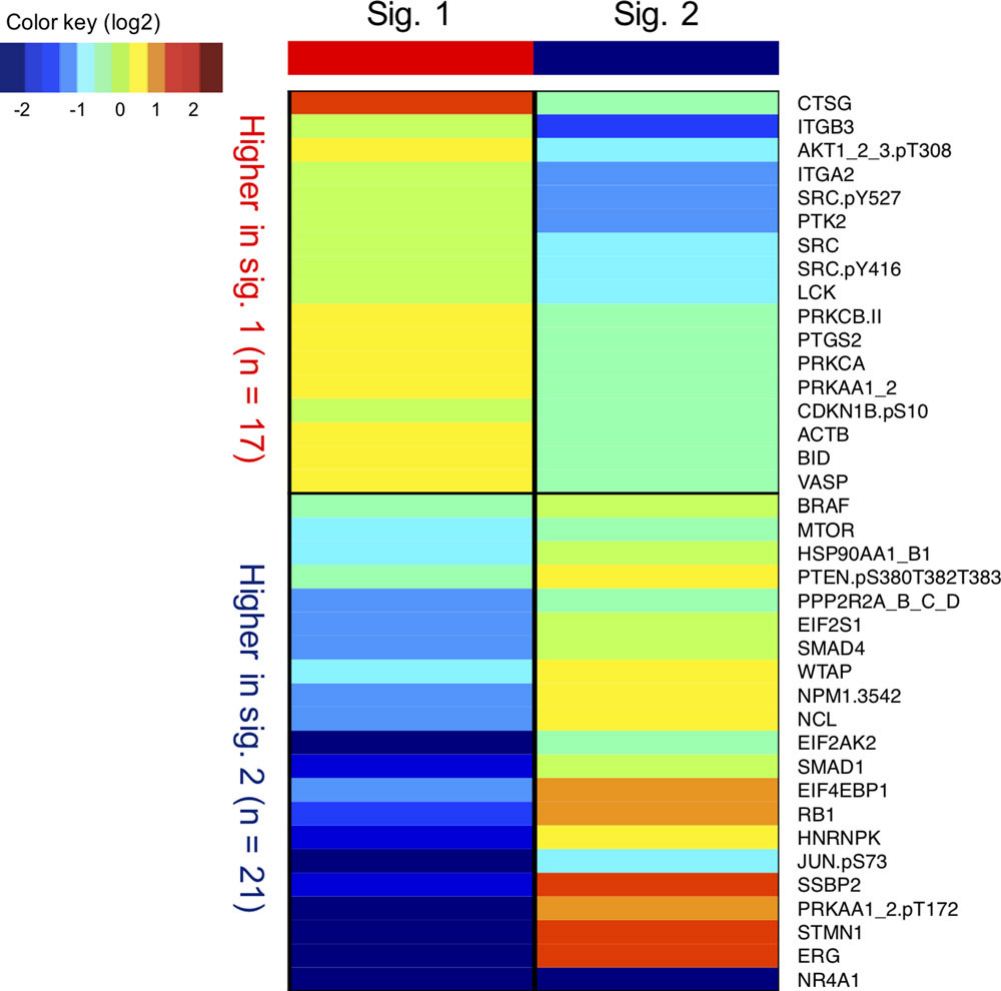
Heat map showing the differentially expressed proteins between APL S1 and S2. Seventeen proteins were upregulated in S1 and 21 proteins were upregulated in S2. Colors reflect the median expression levels relative to the healthy CD34+ cells, ranging from low (dark blue) to high (red).

Direct comparison of protein expression levels between the two signatures showed 38 differentially expressed proteins (Figure 4). Seventeen of those proteins had a higher expression in S1, including ACTB, AKT1/2/3.pT308, BID, CDKN1B.pS10, CTSG, ITGA2, ITGB3, LCK, PRKAA1/2, PRKCA, PRKCB.II, PTGS2, PTK2, SRC, SRC.pY416, SRC.pY527, and VASP. The other 21 proteins had a generally higher expression in S2: BRAF, EIF2AK2, EIF2S1, EIF4EBP1, ERG, HNRNPK, HSP90AA1/B1, JUN.pS73, MTOR, NCL, NPM1, NR4A1, PPR2A/B/C/D, PRKAA1/2.pT172, PTEN.pS380T382, RB1, SMAD1, SMAD4, STMN1, SSBP2, and WTAP. To assess the relation between the proteins that characterized the protein signatures, pathway analysis was applied using STRING 10.5. If an antibody was not specific to one protein member (e.g., HSPAA1_B1, PRKAA1_2) a representative member was selected and included in the analysis. We created two sets of proteins, one set associated with S1 and one set associated with S2. This resulted in significant enrichments with proteins in S1 in pathways including platelet activation (*p* = 8.43E‐9), vascular endothelial growth factor receptor signaling (*p* = 8.43E‐9), and transmembrane receptor protein tyrosine kinase signaling pathway (*p* = 1.15E‐8). Proteins associated with S2 were significantly enriched for cellular component biogenesis (*p* = 4.79E‐8), regulation of gene expression (*p* = 2.05E‐6), signal transduction (*p* = 1.39E‐5), transcription from RNA polymerase II promoter (*p* = 2.80E‐5), and RNA metabolic processes (*p* = 8.44E‐5) (Table S4, Supporting Information, for the full list of enrichments). Given that we have previously identified differential expression of heterogeneous nuclear ribonucleoprotein K (HNRNPK) as being prognostic in AML,^26^, ^27^ we sought to validate its differential expression by performing immunohistochemistry analysis. IHC confirmed variable expression in APL, with higher HNRNPK expression in S2 patients, correlating with the RPPA results (Figure S2, Supporting Information).

## 4 Discussion

Although APL is the most favorable form of AML, it still remains a life‐threating disease for a subset of high‐risk APL patients, which indicates that there is a need to be able to identify these patients and to develop additional therapies for them. In this study, we aimed to identify protein expression levels and protein profiles using RPPA from primary APL samples and to compare protein expression seen in APL to those observed in non‐APL AML. We found that despite global overlap between APL and AML, which may be because AML and APL are both malignant myeloid hematological diseases and thus sharing many pathways, approximately 10% of the protein expression levels in APL were significantly different when compared to AML. Most proteins that were higher expressed in APL were involved in the pro‐apoptotic pathway (e.g., BCL2, BCL2L1, DIABLO, YAP1p, YWHAZ) or were linked to a higher proliferation (e.g., CDKN2A, PIK3CA), which may suggest a more pro‐apoptotic tendency of those cells, and that once the differentiation block is abrogated by the ATRA treatment, those cells tend to be more sensitive to the ATO‐induced apoptosis. Proteins that were higher expressed in non‐APL AML were also involved in pro‐proliferative regulation (e.g., GSK3A_B, KDR), suggesting that all AML subtypes were characterized by an increased proliferation rate, though via slightly different mechanisms.

Analysis of the 20 APL patients separately from the non‐APL AML cases discerned two signatures solely based on their proteomic profiles and, thus, irrespective of mutant FLT3 status or cytogenetic aberrations that yielded prognostic information. S2 had a nearly significant higher relapse rate with all the relapse patients clustered within this signature. A selection of those differentially expressed proteins were also previously identified by Harris et al. as being ATRA responsive, either induced or suppressed. In their study, they found that upon treatment, proteins involved in translation initiation and elongation were significantly down regulated, as well as down regulation of heterogeneous nuclear ribonucleoproteins and the protein phosphatase 2A (PP2A), suggesting that post‐transcriptional suppressive pathways are activated during ATRA‐induced growth inhibition and differentiation processes in APL.^28^ Liu et al. selected a multidrug‐resistant leukemia cell line (HL‐60[R]) and a non‐resistant cell line, which they exposed to ATRA, followed by a sequential increase of the concentration.^29^ They found differential expression of genes involved in oxidative phosphorylation and metabolism in HL‐60[R] cells, as well as upregulation of genes involved in protein synthesis, such as eukaryotic translation initiation factors, transcription and elongation factors, and splicing factors. In our samples, taken at the time of diagnosis, we observed higher expression in S2 of the translation and elongation factors EIF2S1, EIF2AK2, EIF4EBP1, as well as of the heterogeneous hnRNP K, an important protein that is known to bind to and regulate the expression of various eIF genes.^26^, ^27^ EIF2AK2 is a protein kinase that, in its activated form, can phosphorylate (activate) the translation initiation factor EIF2S1, which in turn acts as an inhibitor of its own subunit EIF2B with the consequence that translation comes to a halt. Notably, EIF2S1 phosphorylation on serine domain 51 was indeed strongly expressed in S2 compared to normal CD34+ cells. In addition, EIF4EBP1 is a member of the translation repressor proteins and only in its phosphorylated form can EIF4EBP1 dissociate from EIF4E and in turn activate the cap‐dependent mRNA translation. Therefore, upregulation of those three proteins may suggest lower baseline activity of protein synthesis in S2 compared to S1. As in neoplastic cell ribosome biogenesis, translation initiation and elongation processes are increased to sustain the high proliferation rate, and ribosome composition is altered to modulate specific gene expression driving tumorigenesis. This may be associated with a lower proliferation rate in the more chemoresistant patients (higher frequency of relapse) that formed S2 based on their expression patterns. Additionally, we observed lower levels of LCK, SRC, PIK3CA, CDKN1B.pS10 (p27), and upregulation of RB1 (active, non‐phosphorylated) in S2, which also suggest a decreased proliferation rate in S2. Because cells that proliferate less are cycling less, they might not receive as much input through interaction with the stromal cells as highly cycling cells do, which again may contribute to more chemoresistance. Previously, Radu et al. also reported phosphorylation at the serine 10 (S10) residue of CDKN1B as an important event in mediating a role of CDKN1B in ATRA‐induced growth arrest in ovarian carcinoma cell lines.^30^ Phosphorylation at S10 increases the stability of CDKN1B and signals the nuclear export of CDKN1B to the cytoplasm upon cell cycle re‐entry. When they created a mutant form by replacing serine by alanine so that CDKN1B could not be phosphorylated anymore at S10, they saw that cells were more resistant to therapy. In ATRA‐sensitive CAOV3 cells, they also found an increase in the level of S10 phosphorylation of CDKN1B upon treatment. Together, this suggested that phosphorylation of S10 of CDKN1B was critical for inhibition of growth by ATRA. Furthermore, we found three proteins that were part of the histone modification core in S2, NCL, NPM1.3542, and WTAP. Abnormal expression of histone modulators leads to aberrant gene expression and could contribute to leukemogenesis via misregulation of gene transcription of tumor suppressor genes and oncogenes. Previously, our group already found tight correlations in the 205 adult AML patients that were also part of this study between KDM1A, HNRNPK, NCL, SIRT1, ASH2L, and WTAP.^9^ In that study, we observed that patients that expressed high levels of KDM1A, HNRNPK, NCL, SIRT1, ASH2L, and WTAP expressed lower levels of correlated CBL, LCK, SRC phosphorylated on tyrosine 416 and 452, PTK2, ITGA2, SRC, PTG2S, PIK3AC, and FN1, which was again replicated by our observations here.

In conclusion, these data suggest that high‐risk APL patients could be identified and stratified from low‐risk APL patients based on their protein expression patterns. Additionally, these findings provide a potential explanation as to why some patients are more resistant to therapy than others that can be examined in the laboratory. The next step would be to identify a panel of a limited number of proteins that could relatively quickly determine to which signature a new patient belongs at the time of diagnosis and be implemented during risk stratification. By knowing the signature membership of individual APL patients at an early stage, we could then decide whether or not additional therapies are required, based on their high‐ or low‐risk protein profiles. However, to test whether this classification could be used for real purposes, validation is required in a larger independent cohort of APL patients and a broader number of antibodies that cover the pathways that were studied with more depth. A complete and in‐depth proteomic analysis (e.g., 2DE‐DIGE or iTRAQ LC‐MS/MS) on APL protein extracts can guide to identify differential proteins that would indicate which antibodies to use. Another clinical application could be to select drugs that target aberrant protein patterns in combination with standard therapy in high‐risk patients. For instance, S2 was linked to higher levels of BRAF, HSP90AA1_B1, PPP2R2A_B_C_D, and PTEN.pS380T382T383 proteins that are all potentially targetable. However, further research is needed to test those combinations of drugs.

## Conflict of Interest

The authors declare no conflicts of interest.

## Supporting information

Supporting Information

Supporting Information

Supporting Information

Supporting Information

Supporting Information

Supporting Information
