# Supplementary material for: Proteomic Profiling of Acute Promyelocytic Leukemia Identifies Two Protein Signatures Associated with Relapse
Source: Proteomics Clin Appl. 2019 Feb 4;13(4):1800133. doi: 10.1002/prca.201800133 (PMC6635093; doi:10.1002/prca.201800133)
Supplement: Supplementary file 1 — Supporting Information [file PRCA-13-na-s001.pdf]

Supplementary Figures

A B C

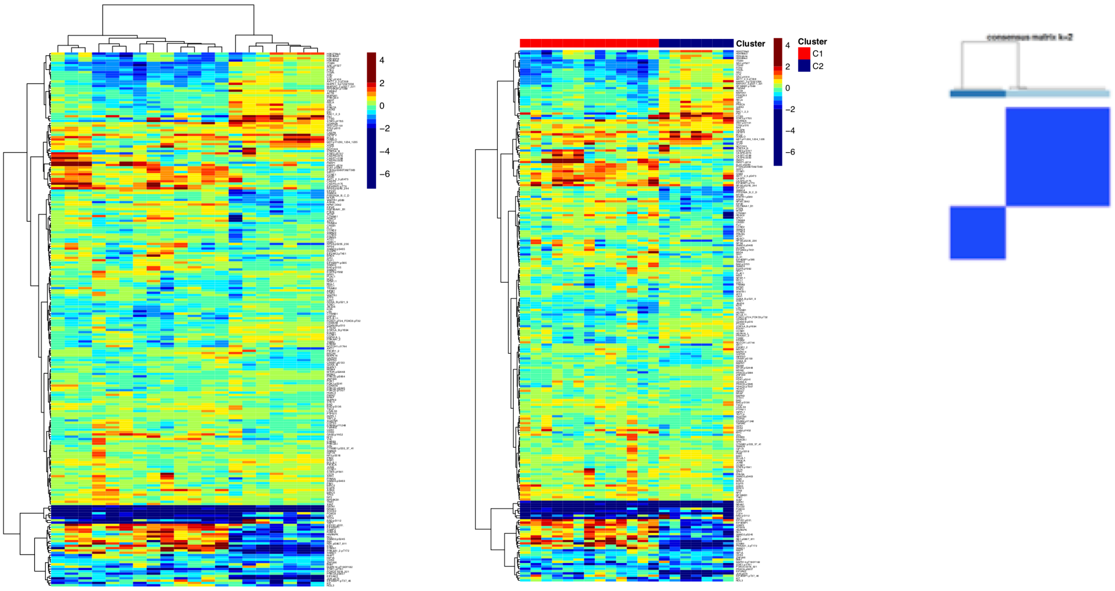

Supplementary Figure S1: In this figure we show the robustness of the clustering using three different clustering methods: unsupervised hierarchical clustering (**A**), progeny clustering (**B**), and the consensus clustering (**C**). Each method identified an identical division of the 20 APL patients into two distinct protein signatures.

A B

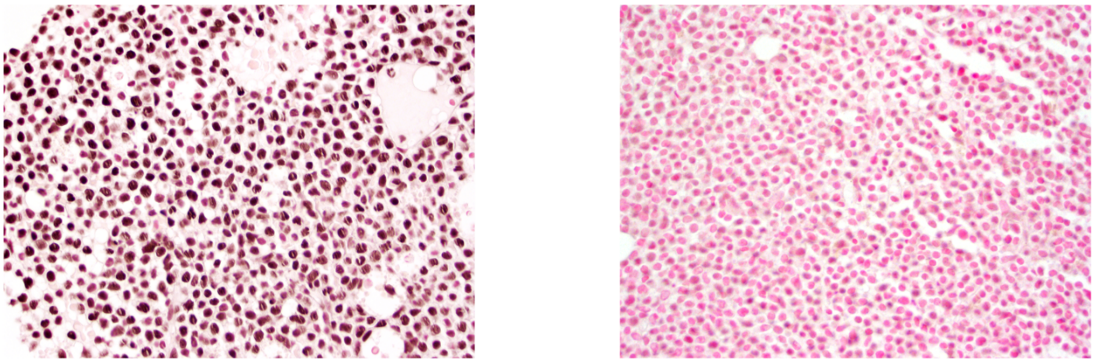

RPPA: 1.23438 (log2) RPPA: -0.32467 (log2)

Supplementary Figure S2: This figure shows immunohistochemistry (IHC) analysis for two APL bone marrow biopsies that correspond to samples used in the RPPA analysis. It shows variable expression between the two samples, that strongly correlates with the RPPA results. (**A**) High hnRNP K and (**B**) low hnRNP K expression assessed by both IHC and RPPA.
