# Supplementary material for: Proteomic Profiling of Acute Promyelocytic Leukemia Identifies Two Protein Signatures Associated with Relapse
Source: Proteomics Clin Appl. 2019 Feb 4;13(4):1800133. doi: 10.1002/prca.201800133 (PMC6635093; doi:10.1002/prca.201800133)
Supplement: Supplementary file 2 — Supporting Information [file PRCA-13-na-s002.pdf]

Variable category	All
**Number of cases (n)**	25
**Male**	49.3%
**Age, y**	
Mean	59.1
SD	15.6
**FAB**	
M0	5.9%
M1	12.7%
M2	33.2%
M4	28.3%
M5	12.2%
M6	2.0%
M7	2.4%
RAEBT	2.9%
NA	50.0%
**Cytogenetics**	
Favorable	10.7%
Intermediate	46.3%
Unfavorable	42.9%
**FLT3 ITD**	
N (tested)	95.6%
Mutant	23.0%
Wildtype	77.0%
**FLT3 D835**	
N (tested)	95.6%
Mutant	9.7%
Wildtype	90.3%
**FLT3**	
N (tested)	95.6%
Mutant	29.6%
Wildtype	70.4%
**NPM1**	
N (tested)	80.5%
Mutant	22.4%
Wildtype	77.6%
