# Supplementary material for: Proteomic Profiling of Acute Promyelocytic Leukemia Identifies Two Protein Signatures Associated with Relapse
Source: Proteomics Clin Appl. 2019 Feb 4;13(4):1800133. doi: 10.1002/prca.201800133 (PMC6635093; doi:10.1002/prca.201800133)
Supplement: Supplementary file 3 — Supporting Information [file PRCA-13-na-s003.pdf]

Protein Name Rosetta Stone					RPPA Staining Details						Functional Category
RPPA Antibody Name	Huge Name (added with PTMs)	MiMI Name	Full Name/Description from GeneCards	Functional Effect of Phosphorylation	Common Name Mfg Claimed Target	Manufacturer	Catalog#	Anti-body Source	Anti-body Dilution	2nd Ab dilution	Functional Group
ACTB	ACTB	ACTB	Actin Beta		Actin(β)	Sigma	A5441	mouse	2000	20000	cytoskeletal
AIFM1	AIFM1	AIFM1	apoptosis-inducing factor, mitochondrion-associated, 1		AIF	Santa Cruz	sc-13116	mouse	250		ApopReg
AKT1	AKT1	AKT1	v-akt murine thymoma viral oncogene homolog 1		AKT1	Cell Signaling	9272	Rabbit	150	15000	pi3kakt
AKT1_2_3.pS473	AKT1/AKT2/AKT3-phospho ser473	AKT1	v-akt murine thymoma viral oncogene homolog 1	Activation	AKT-P473(Ser)	Cell Signaling	9271	Rabbit	50	15000	pi3kakt
AKT1_2_3.pT308	AKT1/AKT2/AKT3-phospho Thr308	AKT1	v-akt murine thymoma viral oncogene homolog 1	Activation	AKT-P308(Thr)	Cell Signaling	9275	Rabbit	50	15000	pi3kakt
ASH2L	ASH2L	ASH2L	ash2 (absent, small, or homeotic)-like (Drosophila)		Ash2L	Cell Signaling	5019	Rabbit	5000	15000	histone
ASNS	ASNS	ASNS	asparagine synthetase (glutamine-hydrolyzing)		ASNS	Sigma	HPA029 318	Rabbit	500	15000	metabolic
ATF3	ATF3	ATF3	activating transcription factor 3		ATF3	Abcam	ab87213	Rabbit	500	15000	autophagy,creb
ATG7	ATG7	ATG7	autophagy related 7		ATG7	Cell Signaling	8558	Rabbit	500	15000	autophagy
BAD	BAD	BAD	BCL2-associated agonist of cell death		Bad	Cell Signaling	9292	Rabbit	100	15000	BH3
BAD.pS112	BAD-phospho Ser112	BAD	BCL2-associated agonist of cell death	Inactivation	Bad-p112(Ser)	Cell Signaling	9291	Rabbit	100	15000	BH3
BAD.pS136	BAD-phospho Ser136	BAD	BCL2-associated agonist of cell death	Inactivation	Bad-p136(Ser)	Cell Signaling	9295	Rabbit	50	15000	BH3
BAD.pS155	BAD-phospho Ser155	BAD	BCL2-associated agonist of cell death	Inactivation	Bad-p155(Ser)	Cell Signaling	9297	Rabbit	100	15000	BH3
BAK1	BAK1	BAK1	BCL2-antagonist/killer 1		Bak	Cell Signaling	3792	Rabbit	50	15000	BH3
BAX	BAX	BAX	BCL2-associated X protein		Bax	Cell Signaling	2772	Rabbit	100	15000	BH3
BCL2	BCL2	BCL2	B-cell CLL/lymphoma 2		Bcl2	DAKO	M0887	mouse	200	15000	BH3
BCL2L1	BCL2L1	BCL2L1	BCL2-like 1		Bcl-XL	Cell Signaling	2762	Rabbit	500	20000	BH3
BCL2L11	BCL2L11	BCL2L11	BCL2-like 11 (apoptosis facilitator)		Bim	Epitomics	1036-1	Rabbit	200	15000	BH3
BECN1	BECN1	BECN1	beclin 1, autophagy related		Beclin-1	Cell Signaling	3738	Rabbit	500	15000	autophagy
BID	BID	BID	BH3 interacting domain death agonist		Bid	Cell Signaling	2002	Rabbit	250	15000	BH3
BIRC2	BIRC2	BIRC2	baculoviral IAP repeat containing 2		CIAP-1	Upstate	07-759	Rabbit	200	15000	IAP
BIRC5	BIRC5	BIRC5	baculoviral IAP repeat containing 5		Survivin	Cell Signaling	2802	Rabbit	50	15000	IAP
BMI1	BMI1	BMI1	*BMI1* polycomb ring finger oncogene		Bmi-1	Cell Signaling	2830	Rabbit	150	15000	histone
BRAF	BRAF	BRAF	v-raf murine sarcoma viral oncogene homolog B		Raf-B	Santa Cruz	sc5284	mouse	100	15000	mek

BRD4	BRD4	BRD4			BRD4	Epitomics	5716-1	Rabbit	250	15000	histone
CASP3	CASP3	CASP3	caspase 3, apoptosis-related cysteine peptidase		caspase 3	Cell Signaling	9662	Rabbit	250	15000	ApopOccur
CASP3.cl175	CASP3 cleaved	CASP3	caspase 3, apoptosis-related cysteine peptidase		Caspase 3 cleaved Asp175	Cell Signaling	9661	Rabbit	100	15000	ApopOccur
CASP7.cl198	CASP7 cleaved Asp198	CASP7	caspase 7, apoptosis-related cysteine peptidase		Caspase 7 cleaved Asp198	Cell Signaling	9491	Rabbit	250	15000	ApopOccur
CASP8	CASP8	CASP8	caspase 8, apoptosis-related cysteine peptidase		Caspase 8	Cell Signaling	9746	mouse	250	15000	ApopOccur
CASP9.cl315	CASP9 cleaved Asp315	CASP9	caspase 9, apoptosis-related cysteine peptidase		Caspase 9 cleaved Asp315	Cell Signaling	9505	Rabbit	250	15000	ApopOccur
CASP9.cl330	CASP9 cleaved Asp330	CASP9	caspase 9, apoptosis-related cysteine peptidase		Caspase 9 cleaved Asp330	Cell Signaling	9501	Rabbit	250	15000	ApopOccur
CAV1	CAV1	CAV1	caveolin 1, caveolae protein, 22kDa		Caveolin-1	Cell Signaling	3238	Rabbit	100	15000	adhesion
CBL	CBL	CBL	*Cbl* proto-oncogene, E3 ubiquitin protein ligase		Cbl-c	BD sciences	610441	mouse	1000	15000	ubiquitin
CCNB1	CCNB1	CCNB1	cyclin B1		Cyclin B1	Santa Cruz	SC245	mouse	100	15000	cellcycle
CCND1	CCND1	CCND1	cyclin D1		Cyclin D1(M-20)	Santa Cruz	sc718	Rabbit	500	15000	cellcycle
CCND3	CCND3	CCND3	cyclin D3		Cyclin D3	Cell Signaling	2936	mouse	100	15000	cellcycle
CCNE1	CCNE1	CCNE1	cyclin E1		Cyclin E	Santa Cruz	sc-247	Rabbit	100	15000	cellcycle
CCNE2	CCNE2	CCNE2	cyclin E2		Cyclin E2	Cell Signaling	1142	Rabbit	250	15000	cellcycle
CD44	CD44	CD44	*CD44* molecule (Indian blood group)		CD44.Epi	Epitomics	1998-1	Rabbit	2000	15000	adhesion
CD74	CD74	CD74	*CD74* molecule, major histocompatibility complex, class II invariant chain		CD74	Santa Cruz	sc-6262	mouse	300	15000	tcell
CDK1	CDK1	CDC2	cyclin-dependent kinase 1		CDC2	calbiochem	cc01	mouse	200	15000	cellcycle
CDK2	CDK2	CDK2	cyclin-dependent kinase 2		CDK2	Santa Cruz	SC6248		200	15000	cellcycle
CDK4	CDK4	CDK4	cyclin-dependent kinase 4		CDK4	Cell Signaling	2906	mouse	200	15000	cellcycle
CDKN1A	CDKN1A	CDKN1A	cyclin-dependent kinase inhibitor 1A (p21, Cip1)		P21/Waf	Cell Signaling	2946	mouse	250	10000	cellcycle
CDKN1B	CDKN1B	CDKN1B	cyclin-dependent kinase inhibitor 1B (p27, Kip1)		P27	Santa Cruz	sc-528	Rabbit	250	15000	cellcycle
CDKN1B.pS10	CDKN1B PhosphoSer10	CDKN1B	cyclin-dependent kinase inhibitor 1B (p27, Kip1)	Apoptosis altered	P27ps10	Epitomics	2187-1	Rabbit	500	15000	cellcycle
CDKN2A	CDKN2A	CDKN2A	cyclin-dependent kinase inhibitor 2A		P16	santa Cruz	sc468	Rabbit	5000	15000	cellcycle
CLPP	CLPP	CLPP	caseinolytic mitochondrial matrix peptidase proteolytic subunit		CLPP	Abcam	124822	Rabbit	1000	15000	histone
COPS5	COPS5	COPS5	COP9 signalosome subunit 5		JAB1	Santa Cruz	sc-13157	mouse	300	15000	ubiquitin
CREB1	CREB1	CREB1	cAMP responsive element binding protein 1		CREB	Epitomics	1496-1	Rabbit	2000	15000	creb
CREB1.pS133	CREB2 phospho Ser133	CREB1	cAMP responsive element binding protein 1	Activation	CREB-p(ser133)	Epitomics	1113-1	Rabbit	2000	15000	creb
CTNNA1	CTNNA1	CTNNA1	catenin (cadherin-associated protein), alpha 1, 102kDa		Catenin-alpha	calbiochem	CA1030	mouse	75	15000	wnt

CTNNB1	CTNNB1	CTNNB1	catenin (cadherin-associated protein), beta 1, 88kDa		Catenin-beta	Cell Signaling	9562	Rabbit	50	15000	wnt
CTNNB1.p S33_37_41	CTNNB1-phospho Ser33/37/Thr41	CTNNB1	catenin (cadherin-associated protein), beta 1, 88kDa	Leads to degredati on	catenin-beta phospho-	Cell Signaling	9561	Rabbit	500	15000	wnt
CTSG	CTSG	CTSG	cathepsin G		CTSG	Abcam	ab8816	sheep	500	30000	adhesion
DIABLO	DIABLO	DIABLO	*blo*, IAP-binding mitochondrial protein		Smac/Diablo	Cell Signaling	2954	mouse	500	15000	IAP
DLX1	DLX1	DLX1	distal-less homeobox 1		DLX1	Abnova	H000017 45-M01	mouse	1000	15000	transcription
DUSP6	DUSP6	DUSP6	dual specificity phosphatase 6		DUSP6	abcam	ab76310	Rabbit	3000	15000	mek
EGFR	EGFR	EGFR	Epiidermal growth factor receptor		EGFR	Santa Cruz	sc-03	Rabbit	500	20000	mtor,STP
EGFR.pY99 2	EGFR-phospho Tyr992	EGFR	Epiidermal growth factor receptor	Activation	EGFR-p tyr992	Cell Signaling	2235	Rabbit	50	15000	mtor
EGLN1	EGLN1	EGLN1	egl-9 family hypoxia-inducible factor 1		Egln1	Millipore	05-1327	mouse	500	5000	hypoxia
EIF2AK2	EIF2AK2	EIF2AK2	ryotic translation initiation factor 2-alpha kinase 2		PRKR(EIF2AK 2)	Abnova	H000056 10-M02	mouse	5000	15000	transcription
EIF2AK2.pT 451	EIF2AK2.phospho Thr 451	EIF2AK2	ryotic translation initiation factor 2-alpha kinase 2	Activation	PRKRpTh451(EIF2AK2)	invitrogen	44-668G	Rabbit	1500	15000	transcription
EIF2S1	EIF2S1	EIF2S1	eukaryotic translation initiation factor 2, subunit 1 alpha, 35kDa		eIF2	Cell Signaling	9722	Rabbit	3000	15000	transcription
EIF2S1.pS5 1.	EIF2S1-phospho Ser51	EIF2S1	eukaryotic translation initiation factor 2, subunit 1 alpha, 35kDa	Stabalizes	phospho-eIF2-alpha	Cell Signaling	9721	Rabbit	250	15000	transcription
EIF4E	EIF4E	EIF4E	otic translation initiation factor 4E		eIF4E	Cell Signaling	9742	Rabbit	200	15000	transcription
EIF4EBP1	EIF4EBP1	EIF4EBP1	proliferation-associated 2G4, 38kDa		EBP1	Cell Signaling	9452	Rabbit	500	15000	s6rp
EIF4EBP1. pS65	EIF4EBP1.phospho Ser S65	EIF4EBP1	proliferation-associated 2G4, 38kDa	Inactivatio n	EBP1.pser65	Cell Signaling	9456	Rabbit	400	15000	s6rp
EIF4EBP1. pT37_46	EIF4EBP1.phospho Thr T37 & 46	EIF4EBP1	proliferation-associated 2G4, 38kDa	Inactivatio n	EBP1.pthr37.4 6	Cell Signaling	9459	Rabbit	1000	15000	s6rp
EIF4EBP1. pT70	EIF4EBP1.phospho Thr T70	EIF4EBP1	proliferation-associated 2G4, 38kDa	Inactivatio n	EBP1.pthr70	Cell Signaling	9455	Rabbit	200	15000	s6rp
ELK1.pS38 3	ELK1-phospho Ser383	ELK1	*ELK1*, member of ETS oncogene family	Activation	Elk(phospho-ser383)	Cell Signaling	9181	Rabbit	100	15000	mek,pi3kakt
ERBB2	ERBB2	ERBB2	v-erb-b2 avian erythroblastic leukemia viral oncogene homolog 2		HER2/Erb2	Cell Signaling	2242	Rabbit	250	15000	STP
ERBB2.pY1 248	ERBB2-phospho Tyr1248	ERBB2	v-erb-b2 avian erythroblastic leukemia viral oncogene homolog 2	Activation	HER2(p-Tyr1248)	Upstate	06-229	Rabbit	1500	15000	STP
ERBB3	ERBB3	ERBB3	v-erb-b2 avian erythroblastic leukemia viral oncogene homolog 3		HER3	Upstate	05-390	mouse	500	15000	STP
ERG	ERG	EGR1	early growth response 1		ERG1/2/3	Santa Cruz	sc-353	Rabbit	1000	15000	transcription
FLI1	Fli1	FLI1	Fli-1 proto-oncogene, ETS transcription factor		Fli	Dennis Watson		Rabbit	2000	15000	fli1,histone
FN1	FN1	FN1	fibronectin 1		Fibronectin	Epitomics	1574	Rabbit	3000 0	15000	adhesion
FOXO1.pT2 4_FOXO3.p T32	FOXO1-phospho thr24/FOXO3-phospho thr32	FOXO1	forkhead box O1	Inactivatio n	FoxO1a/3a	Cell Signaling	9464	Rabbit	500	15000	cellcycle,pi3kakt
FOXO3	FOXO3	FOXO3	forkhead box O3		FoxO3a	Cell Signaling	9467	Rabbit	500	20000	cellcycle,pi3kakt

FOXO3.S318_321	FOXO3-phospho Ser318/321	FOXO3	forkhead box O3	Inactivation	FKHRL1/FoxO3a (P-Ser 318/321)	Cell Signaling	9465	Rabbit	10000	15000	cellcycle,pi3kakt
GAB2	GAB2	GAB2	GRB2-associated binding protein 2		Gab2	Cell Signaling	3239	Rabbit	500	15000	pi3kakt
GAB2.pY452	GAB2-phospho Tyr452	GAB2	GRB2-associated binding protein 2	Tyr452 is a potential binding site of p85, the regulatory subunit of PI3 kinase.	Gab2-pTyr452	Cell Signaling	3882	Rabbit	100	15000	pi3kakt
GAPDH	GAPDH	GAPDH	glyceraldehyde-3-phosphate dehydrogenase		GAPDH	Cell Signaling	4300	mouse	2000	15000	metabolic
GATA1	GATA1	GATA1	GATA binding protein 1 (globin transcription factor 1)		GATA-1	Cell Signaling	3535	Rabbit	1000	15000	differentiation,transcription
GATA3	GATA3	GATA3	GATA binding protein 3		Gata3	BD bioscience	558686	mouse	500	15000	differentiation,transcription
GSKA_B	GSKA/GSKB	GSK3A	glycogen synthase kinase 3 alpha		GSK3	santa Cruz	sc-7291	mouse	200	15000	autophagy,pi3kakt
GSKA_B.pS21_9	GSKA/GSKB-phospho Ser21/9	GSK3A	glycogen synthase kinase 3 alpha	Inactivation	GSK3a/B(p-ser21/9)	Cell Signaling	9331	Rabbit	200	15000	autophagy,pi3kakt
H3histone	HIST3H3	HIST3H3	*histone* cluster *3*, H3		H3histone	active motif	39163	Rabbit	1500000	40000	histone
H3K27Me3	HIST3H3-K27-Me3	HIST3H3	*histone* cluster *3*, H3 , Lysine 27 methylation 3	Repression	H3K27Me3	active motif	61017	mouse	3000	40000	histone
H3K4Me2	HIST3H3-K4-Me2	HIST3H3	*histone* cluster *3*, H3 , Lysine 4 methylation 2	Repression	H3K4Me2	active motif	39141	Rabbit	8000	20000	histone
H3K4Me3	HIST3H3-K4-Me3	HIST3H3	*histone* cluster *3*, H3 , Lysine 4 methylation 3	Repression	H3K4Me3	active motif	39159	Rabbit	7000	20000	histone
HDAC1	HDAC1	HDAC1	histone deacetylase 1		HDAC1	Imgenex	IM-337	Rabbit	400	20000	histone
HDAC2	HDAC2	HDAC2	histone deacetylase 2		HDAC2	Santa Cruz	sc-7899	Rabbit	1000	15000	histone
HDAC3	HDAC3	HDAC3	histone deacetylase 3		HDAC3	Cell Signaling	2632	Rabbit	100	15000	histone
HIF1A	HIF1A	HIF1A	hypoxia inducible factor 1, alpha subunit (basic helix-loop-helix transcription factor)		HIF-1α	BD pharmingen	610959	mouse	50	15000	hypoxia
HNRNPK	HNRNPK	HNRPK	rogeneous nuclear ribonucleoprotein K		hnRNPK	Santa Cruz	sc-28380	mouse	5000	15000	histone
HSP90AA1_B1	HSP90AA1/HSP90AB1	HSP90AA1	heat shock protein 90kDa alpha (cytosolic), class A member 1		HSP90	Cell Signaling	4875	Rabbit	500	15000	heatshock
HSPA1A_L	HSPA1A/HSPA1L	HSPA1A	heat shock 70kDa protein 1A		HSP70	Cell Signaling	4872	Rabbit	250	15000	heatshock
HSPB1	HSPB1	HSPB1	heat shock 27kDa protein 1		HSP27	Cell Signaling	2402	mouse	100	15000	heatshock
IGF1R	IGF1R	IGF1R	insulin-like growth factor 1 receptor		IGF-1 receptor beta	Cell Signaling	3027	Rabbit	1000	15000	STP
IGFBP2	IGFBP2	IGFBP2	insulin-like growth factor binding protein 2, 36kDa		IGFBP-2	Cell Signaling	3922	Rabbit	250	15000	adhesion
INPP5D	INPP5D	INPP5D	inositol polyphosphate-5-phosphatase, 145kDa		SHIP1	Santa Cruz	SC-8425	mouse	250	15000	mtor
INPPL1	INPPL1	INPPL1	inositol polyphosphate phosphatase-like 1		SHIP2	Cell Signaling	2730	Rabbit	300	15000	mtor

IRS1.pS1101	IRS1-phospho ser 1101	IRS1	insulin receptor substrate 1	Inhibition	IRS-1-p(ser1101)	Cell Signaling	2385	Rabbit	250	15000	mtor,tp53
ITGA2	ITGA2	ITGA2	integrin, alpha 2 (CD49B, alpha 2 subunit of VLA-2 receptor)		CD49b	BD Transduction Lab	611016	mouse	500	15000	adhesion
ITGAL	ITGAL	ITGAL	integrin, alpha L (antigen CD11A (p180), lymphocyte function-associated antigen 1; alpha polypeptide)		CD11a	BD Transduction Lab	610826	mouse	500	15000	adhesion
ITGB3	ITGB3	ITGB3	integrin, beta 3 (platelet glycoprotein IIIa, antigen CD61)		Integrin-beta3	Cell Signaling	4702	Rabbit	250	15000	adhesion
JMJD6	JMJD6	JMJD6	jumonji domain containing 6		JMJD6	Abcam	ab50720	Rabbit	1000	20000	histone
JUN.pS73	JUN-phospho Ser73	JUN	*jun* proto-oncogene	Activation	Jun-C-phospho ser73	Cell Signaling	9164	Rabbit	100	10000	transcription
JUNB	JUNB	JUNB	jun B proto-oncogene		Jun-B	Cell Signaling	3755	Rabbit	100	15000	transcription
KDM1A	KDM1A	KDM1A	lysine (K)-specific demethylase 1A		LSD1	Cell Signaling	2184	Rabbit	3000	15000	histone
KDR	KDR	KDR	kinase insert domain receptor (a type III receptor tyrosine kinase)		VEGFR2	Cell Signaling	2479	Rabbit	700	15000	hypoxia
KIT	KIT	KIT	v-*kit* Hardy-Zuckerman 4 feline sarcoma viral oncogene homolog		Kit-C	Epitomics	1522	Rabbit	1000	15000	mek,STP
LCK	LCK	LCK	lymphocyte-specific protein tyrosine kinase		Lck	Cell Signaling	2752	Rabbit	50	15000	tcell
LEF1	LEF1	LEF1	lymphoid enhancer-binding factor 1		LEF1	Cell Signaling	2230	Rabbit	1000	15000	wnt
LGALS3	LGALS3	LGALS3	lectin, galactoside-binding, soluble, 3		Galectin-3	Santa Cruz	sc-32790	mouse	250	15000	tcell
LYN	LYN	LYN	v-yes-1 Yamaguchi sarcoma viral related oncogene homolog		Lyn	Cell Signaling	2732	Rabbit	250	15000	mek,STP
MAP2K1	MAP2K1	MAP2K1	mitogen-activated protein kinase kinase 1		MEK	Cell Signaling	9122	Rabbit	2000	10000	mapk
MAP2K1_2.pS217_221	MAP2K1/MAP2K2-phospho ser217/221	MAP2K1	mitogen-activated protein kinase kinase 1	Activation	MEK(p-ser217/221)	Cell Signaling	9121	Rabbit	1000	15000	mapk
MAPK1	MAPK1	MAPK1	mitogen-activated protein kinase 1		Erk2	Santa Cruz	Sc-154	Rabbit	2000	15000	mapk
MAPK1_3.pT202Y204	MAPK1/MAPK3-phospho Thr202/Tyr204	MAPK1	mitogen-activated protein kinase 1	Activation	Erk-p42/44(Thr202/Tyr204	Cell Signaling	9101	Rabbit	400	15000	mapk
MAPK14	MAPK14	MAPK14	mitogen-activated protein kinase 14		P38	Cell Signaling	9212	Rabbit	200		mapk
MAPK14.pT180Y182	MAPK14-p180p182	MAPK14	mitogen-activated protein kinase 14	Activation	P38p180p182	Cell Signaling	9211	Rabbit	10	15000	mapk
MAPK9	MAPK9	MAPK9	mitogen-activated protein kinase 9		JNK2	Cell Signaling	4672	Rabbit	25	15000	mapk,stat
MAPT	MAPT	MAPT	microtubule-associated protein tau		Tau	Upstate	05-348	mouse	150	15000	cytoskeletal
MCL1	MCL1	MCL1	myeloid cell leukemia sequence 1 (BCL2-related)		MCL1	BD pharmingen	559027	mouse	50	15000	BH3
MDM2	MDM2	MDM2	*MDM2* oncogene, E3 ubiquitin protein ligase		MDM2	Santa Cruz	sc813	Rabbit	5000	15000	tp53
MDM4	MDM4	MDM4	*Mdm4* p53 binding protein homolog (mouse)		MDM4	Bethly lab	A300-287A	Rabbit	5000	15000	tp53
MET.pY1230_1234_1235	MET-phospho Py1230/1234/1235	MET	*met* proto-oncogene	Activation	C-Met-pTyr 1230/1234/1235	Biosource	44-888G	Rabbit	250	20000	STP

MTOR	MTOR	FRAP1	mechanistic target of rapamycin (serine/threonine kinase)		mTor	Cell Signaling	2983	Rabbit	200	10000	mtor
MTOR.pS2448	MTOR-phospho Ser2448	FRAP1	mechanistic target of rapamycin (serine/threonine kinase)	Activation	mTor(p-Ser2448)	Cell Signaling	2971	Rabbit	100	15000	mtor
MYC	MYC	MYC	v-*myc* avian myelocytomatosis viral oncogene homolog		Myc	Cell Signaling	9402	Rabbit	100	15000	cellcycle,transcription
NCL	NCL	NCL	nucleolin		C23 (nucleolin)	Santa Cruz	sc8031	mouse	200	15000	fli1,histone
NF2	NF2	NF2	neurofibromin 2 (merlin)		NF2	Santa Cruz	sc332	Rabbit	500	15000	cytoskeletal,hippo
NF2.pS518	NF2-phospho ser518	NF2	neurofibromin 2 (merlin)	Inactivation	pNF2(ser518)	chemicon	AB5607	Rabbit	500	15000	cytoskeletal,hippo
NOL3	ARC	ARC	activity-regulated cytoskeleton-associated protein		ARC	Imgenex	IMG-171	Rabbit	2000	15000	ApopReg,BH3
NOTCH1.cl1744	NOTCH1 cleaved val1744	NOTCH1	notch 1	Activation	Notch1-cleaved (Val1744)	Cell Signaling	4147	Rabbit	400	15000	tcell
NOTCH3	NOTCH3	NOTCH3	notch 3		Notch3	santa Cruz	sc5593	Rabbit	200	15000	tcell
NPM1	NPM1	NPM1	nucleophosmin (nucleolar phosphoprotein B23, numatrin)		NPM	invitrogen	32-5200	mouse	10000	15000	histone
NPM1.3542	NPM1mut AA3542	NPM1	nucleophosmin (nucleolar phosphoprotein B23, numatrin)		NPM1.3542	Cell Signaling	3542	Rabbit	1000	15000	fli1
NR4A1	NR4A1	NR4A1	nuclear receptor subfamily 4, group A, member 1		Nur77	Imgenex	IMG-528	Rabbit	200	15000	transcription
NRP1	NRP1	NRP1	neuropilin 1		NRP1(neuropilin)	Santa Cruz	SC-5307	mouse	10	20000	cytoskeletal
ODC1	ODC1	ODC1	ornithine decarboxylase 1		ODC	Shantz/Lisa Lab			500	15000	metabolic
PARK7	PARK7	PARK7	parkinson protein 7		DJ-1	Private-Andreeff		Rabbit	500	15000	autophagy
PARP1	PARP1	PARP1	poly (ADP-ribose) polymerase 1		PARP	Cell Signaling	9542	Rabbit	200	15000	ApopOccur
PARP1.cl214	PARP1-cleaved Asp 214	PARP1	poly (ADP-ribose) polymerase 1	Activation	PARP(cleaved Asp214)	Cell Signaling	9541	Rabbit	100	15000	ApopOccur
PDK1	PDK1	PDK1	pyruvate dehydrogenase kinase, isozyme 1		PDK1	Cell Signaling	3062	Rabbit	200	15000	mtor
PDK1.pS241	PDK1-phospho ser241	PDK1	pyruvate dehydrogenase kinase, isozyme 1	Activation	PDK1-p241(Ser)	Cell Signaling	3061	Rabbit	500	15000	mtor
PIK3CA	PIK3CA	PIK3CA	phosphotidylinositol-4,5-bisphosphate 3-kinase, catalytic subunit alpha		PI3 K p110-alpha	Epitomics	1683-1	Rabbit	200	15000	mtor
PIK3R1_2	PIK3R1/PIK3R2	PIK3R1	phosphoinositide-3-kinase, regulatory subunit 1 (alpha)		PI3 K p85	Cell Signaling	4292	Rabbit	20	15000	mtor
PIM1	PIM1	PIM1	pim-1 oncogene		PIM1	Santa Cruz	sc-13153	mouse	100	15000	differentiation
PIM2	PIM2	PIM2	pim-2 oncogene		PIM2	Cell Signaling	4730	Rabbit	200	15000	differentiation
PPARA	PPARA	PPARA	peroxisome proliferator-activated receptor alpha		PPARA	boster Bio	PA1412	Rabbit	0.5ug/ul	30000	metabolic
PPARG	PPARG	PPARG	peroxisome proliferator-activated receptor gamma		PPARγ	Santa Cruz	sc7273	mouse	75	15000	differentiation
PPP2R2A_B_C_D	PPP2R2D/PP2R2A/PPP2R2B/PPP2R2C	PPP2R4	protein phosphatase 2A activator, regulatory subunit 4		PP2A-B55	Santa Cruz	sc-18330	goat	500	15000	STP
PRKAA1_2	PRKAA1/PRKAA2	PRKAA1	protein kinase, AMP-activated, alpha 1 catalytic subunit		AMPKα	cell signaling	2532	Rabbit	200	15000	autophagy

PRKAA1_2.pT172	PRKAA1/PRKAA2-phospho Thr172	PRKAA1	protein kinase, AMP-activated, alpha 1 catalytic subunit	Activation	AMPKα P(Thr172)	cell signaling	2535	Rabbit	200	15000	autophagy
PRKCA	PRKCA	PRKCA	protein kinase C, alpha		PKCα	Upstate	05-154	mouse	2000	15000	pkc
PRKCA.pS657	PRKCA-phospho ser657	PRKCA	protein kinase C, alpha	Activation	PKCα-p657(Ser)	Upstate	06-822	Rabbit	1000	20000	pkc
PRKCB.I	PRKCB	PRKCB	protein kinase C, beta		PKCβ I	Santa Cruz	sc8049	mouse	300	20000	pkc
PRKCB.II	PRKCB	PRKCB	protein kinase C, beta		PKCβII	Santa Cruz	13149	mouse	200	15000	pkc
PRKCD.pS645	PRKCD-phospho Ser645	PRKCD	protein kinase C, delta		PKCδ-645(Ser)	Upstate	07-875	Rabbit	200	30000	pkc
PRKCD.pS664	PRKCD-phospho Ser664	PRKCD	protein kinase C, delta		PKCδ-p664(Ser)	Upstate	07-874	Rabbit	250	30000	pkc
PRKCD.pT507	PRKCD-phospho Thr507	PRKCD	protein kinase C, delta	Activation	PKCδ-507(Thr)	Santa Cruz	sc11770	Goat	1000	30000	pkc
PTEN	PTEN	PTEN	phosphatase and tensin homolog		PTEN	Upstate	07-016	Rabbit	400	15000	mtor
PTEN.pS380T382T383	PTEN	PTEN	phosphatase and tensin homolog	Inactivation	PTEN-p (380/382/383)	Cell Signaling	9554	Rabbit	500	15000	mtor
PTGS2	PTGS2	PTGS2	prostaglandin-endoperoxide synthase 2 (prostaglandin G/H synthase and cyclooxygenase)		cox-2	Epitomics	2169-1	Rabbit	250	15000	metabolic
PTK2	PTK2	PTK2	protein tyrosine kinase 2		Fak	Cell Signaling	3285	Rabbit	500	15000	adhesion
PTPN11	PTPN11	PTPN11	protein tyrosine phosphatase, non-receptor type 11		SHP-2	Epitomics	1590-1	Rabbit	500	15000	STP
RAC1_2_3	RAC1/RAC2/RAC3	RAC1	ras-related C3 botulinum toxin substrate 1 (rho family, small GTP binding protein *Rac1*)		Rac1/2/3	Cell Signaling	2465	Rabbit	500	15000	mek
RB1	RB1	RB1	retinoblastoma 1		Rb	BD PharMingen	554136	mouse	100	15000	cellcycle
RB1.pS807_811	RB1-phospho ser807/811	RB1	retinoblastoma 1	Inactivation	Rb (P-Ser807/811)	Cell Signaling	9308	Rabbit	250	15000	cellcycle
RELA	RELA	NFKB1	v-rel avian reticuloendotheliosis viral oncogene homolog A		NF-kB p65	Cell Signaling	3034	Rabbit	500	15000	pkc
RPS6	RPS6	RPS6	ribosomal protein S6		S6 Ribosomal protein	Cell Signaling	2217	Rabbit	250	15000	s6rp
RPS6.pS235_236	RPS6-phospho ser235/236	RPS6	ribosomal protein S6	Activation	S6 Ribosomal protein(phospho-ser235/236)	Cell Signaling	2211	Rabbit	1500-2000	15000	s6rp
RPS6.pS240_244	RPS6-phospho ser240/244	RPS6	ribosomal protein S6	Activation	S6 Ribosomal protein(phospho-ser240/244)	Cell Signaling	2215	Rabbit	750-1000	15000	s6rp
RPS6KB1	RPS6KB1	RPS6KB1	ribosomal protein S6 kinase, 70kDa, polypeptide 1		p70S6K	Cell Signaling	9202	Rabbit	250	15000	s6rp
RPS6KB1.pT389	RPS6KB1-phospho thr389	RPS6KB1	ribosomal protein S6 kinase, 70kDa, polypeptide 1	Activation	p70S6K(p-thr389)	Cell Signaling	9205	Rabbit	250	15000	s6rp
SFN	SFN	SFN	stratifin		X14.3.3Sigma	Upstate	05-632	mouse	200	15000	tp53
SIRT1	SIRT1	SIRT1	sirtuin 1		SIRT1	Abcam	ab32441	Rabbit	1000	15000	histone
SMAD1	SMAD1	SMAD1	SMAD family member 1		smad1	Epitomics	1649-1	Rabbit	200	15000	smad
SMAD2	SMAD2	SMAD2	SMAD family member 2		SMAD2	Cell Signaling	5339	Rabbit	5000	15000	smad
SMAD2.pS245	SMAD2.p245	SMAD2	SMAD family member 2	Activation	Smad2-p S245/250/255	Cell Signaling	3104	Rabbit	500	15000	smad

SMAD2.pS465	SMAD2.pS465	SMAD2	SMAD family member 2	Activation	Smad2-p S465/467	Cell Signaling	3108	Rabbit	1000	15000	smad
SMAD3	SMAD3	SMAD3	SMAD family member 3		SMAD3	Cell Signaling	9523	Rabbit	500	15000	smad
SMAD4	SMAD4	SMAD4	SMAD family member 4		smad4	Santa Cruz	sc7966	mouse	1000	15000	smad
SMAD5	SMAD5	SMAD5	SMAD family member 5		SMAD5	epitomics	1682-1	Rabbit	1000	15000	smad
SMAD5.pS463	SMAD5pS463	SMAD5	SMAD family member 5	Activation	Smad5-p S463/465	epitomics	2224-1	Rabbit	500	15000	smad
SMAD6	SMAD6	SMAD6	SMAD family member 6		Smad6	Cell Signaling	9519	Rabbit	100	15000	smad
SOCS2	SOCS2	SOCS2	suppressor of cytokine signaling 2		SOCS2	Abcam	ab92847	Rabbit	200	15000	STP
SPI1	SPI1	SPI1	spleen focus forming virus (SFFV) proviral integration oncogene		PU.1	Cell Signaling	2258	Rabbit	5000	15000	transcription
SPP1	SPP1	SPP1	secreted phosphoprotein 1		Osteopotin	Santa Cruz	sc-21742	mouse	500	15000	adhesion
SQSTM1	SQSTM1	SQSTM1	sequestosome 1		P62	Santa Cruz	sc-28359	mouse	250	15000	autophagy
SRC	SRC	SRC	v-*src* avian sarcoma (Schmidt-Ruppin A-2) viral oncogene homolog		Src	Upstate	05-184	mouse	600	15000	src
SRC.pY416	SRC-phospho tyr416	SRC	v-*src* avian sarcoma (Schmidt-Ruppin A-2) viral oncogene homolog	Activation	Src(phospho-tyr416)	Cell Signaling	2101	Rabbit	400	15000	src
SRC.pY527	SRC-phospho tyr527	SRC	v-*src* avian sarcoma (Schmidt-Ruppin A-2) viral oncogene homolog	Inactivation	Src(phospho-tyr527)	Cell Signaling	2105	Rabbit	100	15000	src
SSBP2	SSBP2	SSBP2	single-stranded DNA binding protein 2		SSBP2-I(alpha)	Private-Nagarajan		Rabbit	1000	15000	differentiation
STAT1	STAT1	STAT1	signal transducer and activator of transcription 1, 91kDa		stat1	Cell Signaling	9172		250	15000	stat
STAT1.pY701	STAT1-phospho tyr701	STAT1	signal transducer and activator of transcription 1, 91kDa	Activation	Stat1(phospho-tyr701)	Cell Signaling	9171	Rabbit	100	15000	stat
STAT3	STAT3	STAT3	signal transducer and activator of transcription 3 (acute-phase response factor)		Stat3	Upstate	06-596	Rabbit	50	15000	stat
STAT3.pS727	STAT3-phospho ser727	STAT3	signal transducer and activator of transcription 3 (acute-phase response factor)	Activation	Stat3-p727(Ser)	Cell Signaling	9134	Rabbit	100	15000	stat
STAT3.pY705	STAT3-phospho tyr705	STAT3	signal transducer and activator of transcription 3 (acute-phase response factor)	Activation	Stat3 p705(Tyr)	Cell Signaling	9131	Rabbit	500	15000	stat
STAT5A_B	STAT5A/STAT5B	STAT5A	signal transducer and activator of transcription 5A		Stat5	Cell Signaling	9352	Rabbit	250	15000	stat
STAT5A_B.pY694	STAT5A/STAT5B phospho Tyr694	STAT5A	signal transducer and activator of transcription 5A	Activation	Stat5(phospho-Tyr694)	Cell Signaling	9351	Rabbit	100	15000	stat
STAT6.pY641	STAT6-phospho tyr641	STAT6	signal transducer and activator of transcription 6, interleukin-4 induced	Activation	Stat6(phospho-tyr641)	Cell Signaling	9361	Rabbit	100	10000	stat
STK11	STK11	STK11	serine/threonine kinase 11		LKB1/STK11	Cell Signaling	3050	Rabbit	500	15000	autophagy
STMN1	STMN1	STMN1	stathmin 1		Stathmin	epitomics	1972-1	Rabbit	30000	15000	fli1

TCF4	TCF4	TCF4	transcription factor 4		TCF-4	Santa Cruz	sc8632	goat	400	15000	transcription
TGM2	TGM2	TGM2	transglutaminase 2		TG2	Abcam	ab2386	mouse	2000	30000	adhesion
TNK1	TNK1	TNK1	tyrosine kinase, non-receptor, 1		TNK1	Agent	AP7722a	Rabbit	400	15000	mek
TP53	TP53	TP53	tumor protein p53		TP53	Cell Signaling	9282	Rabbit	1000	15000	ApopReg,tp53
TP53.pS15	TP53-pS15	TP53	tumor protein p53	Blocks MDM2 binding	TP53(phospho Ser 15)	Cell Signaling	9284	Rabbit	250	15000	ApopReg,tp53
TRIM24	TRIM24	TRIM24	tripartite motif containing 24		TRIM24	Novus	NB100-2597	Rabbit	1000	15000	histone,tp53,ubiquitin
TRIM62	TRIM62	TRIM62	tripartite motif containing 62		TRIM62	from Dr.Quintas-cardama	DEAR1	mouse	150	15000	ubiquitin
TSC2	TSC2	TSC2	tuberous sclerosis 2		TSC2	epitomic	1613-1	Rabbit	500	15000	autophagy,mtor
VASP	VASP	VASP	vasodilator-stimulated phosphoprotein		VASP	Cell Signaling	3112	Rabbit	250	15000	hypoxia
VHL	VHL	VHL	von Hippel-Lindau tumor suppressor, E3 ubiquitin protein ligase		VHL	Novus	NB100-485	Rabbit	2000	15000	hypoxia
WTAP	WTAP	WTAP	Wilms tumor 1 associated protein		WTAP	sanjay bansal	from UTSA	Rabbit	10000	15000	cellcycle,fli1,histone
WWTR1	TAZ	TAZ	WW Domain Containing Transcription Regulator 1/Transcriptional Coactivator With PDZ-Binding Motif		TAZ	Abcam	ab3961	Rabbit	1000	15000	hippo
WWTR1.pS89	TAZ-phospho Ser89	TAZ	WW Domain Containing Transcription Regulator 1/Transcriptional Coactivator With PDZ-Binding Motif	Inactivation	TAZ p-Ser89	Santa Cruz	sc17610R	Rabbit	150	15000	hippo
XIAP	XIAP	BIRC4	X-linked inhibitor of apoptosis		XIAP	Cell Signaling	2042	Rabbit	100	15000	IAP
XPO1	XPO1	XPO1	exportin 1 (CRM1 homolog, yeast)		CRM1	Santa Cruz	sc-5595	Rabbit	2000	15000	cytoskeletal
YAP1	YAP1	YAP1	Yes-associated protein 1		YAP	Cell Signaling	4912	Rabbit	100	15000	ApopReg,hippo
YAP1.p	YAP1 phospho Serine 127	YAP1	Yes-associated protein 1	Inactivation	YAP-p(ser127)	Cell Signaling	4911	Rabbit	500	15000	ApopReg,hippo
YWHAE	YWHAE	YWHAE	tyrosine 3-monooxygenase/tryptophan 5-monooxygenase activation protein, epsilon polypeptide		X14.3.3Epsilon	Santa Cruz	sc-23957	mouse	200	15000	tp53
YWHAZ	YWHAZ	YWHAZ	tyrosine 3-monooxygenase/tryptophan 5-monooxygenase activation protein, zeta polypeptide		14-3-3-zeta	chemicon	AB9746	Rabbit	750	20000	tp53
ZNF296	ZNF296	ZNF296	zinc finger protein 296		ZNF342	abcam	ab51265	Rabbit	1000	15000	transcription
ZNF346	ZNF346	ZNF346	zinc finger protein 346		JAZ111	May-UFL		Rabbit	1000	15000	transcription
